# A trial of patient-oriented problem-solving system for immunology teaching in China: a comparison with dialectic lectures

**DOI:** 10.1186/1472-6920-13-11

**Published:** 2013-01-28

**Authors:** Zhiren Zhang, Wei Liu, Junfeng Han, Sheng Guo, Yuzhang Wu

**Affiliations:** 1 Institute of Immunology, Third Military Medical University of PLA, Gaotanyan Main Street 30, Chongqing, 400038, People's Republic of China

**Keywords:** Patient-oriented problem-solving, Medical education, Immunology, China

## Abstract

**Background:**

The most common teaching method used in China is lecturing, but recently, efforts have been widely undertaken to promote the transition from teacher-centered to student-centered education. The patient-oriented problem-solving (POPS) system is an innovative teaching-learning method that permits students to work in small groups to solve clinical problems, promotes self-learning, encourages clinical reasoning and develops long-lasting memory. To our best knowledge, however, POPS has never been applied in teaching immunology in China. The aim of this study was to develop POPS in teaching immunology and assess students’ and teachers’ perception to POPS.

**Methods:**

321 second-year medical students were divided into two groups: I and II. Group I, comprising 110 students, was taught by POPS, and 16 immunology teachers witnessed the whole teaching process. Group II including the remaining 211 students was taught through traditional lectures. The results of the pre- and post-test of both groups were compared. Group I students and teachers then completed a self-structured feedback questionnaire for analysis before a discussion meeting attended only by the teachers was held.

**Results:**

Significant improvement in the mean difference between the pre- and post-test scores of those in Groups I and II was seen, demonstrating the effectiveness of POPS teaching. Most students responded that POPS facilitates self-learning, helps them to understand topics and creates interest, and 88.12% of students favored POPS over simple lectures. Moreover, while they responded that POPS facilitated student learning better than lectures, teachers pointed out that limited teaching resources would make it difficult for wide POPS application in China.

**Conclusions:**

While POPS can break up the monotony of dialectic lectures and serve as a better teaching method, it may not be feasible for the current educational environment in China. The main reason for this is the relative shortage of teaching resources such as space, library facilities and well-trained teachers.

## Background

Due to the rapid advances in biomedical sciences, biotechnology, information technology, etc., knowledge has grown rapidly in the field of medicine. Accordingly, physicians have been increasingly called upon to use the latest research and technology to diagnose, treat, and prevent disease. Thus, one of the primary goals of medical education is to develop future doctors who can adapt to the conditions of medical practice in a rapidly changing health care environment and maximize healthcare quality [1]. Therefore, medical schools worldwide have undergone various medical education innovations to meet these challenges.

Similarly, medical education in China is undertaking great changes [2]. Owing to the country’s huge population, China’s medical education system is probably the largest in the world. China has 159 institutions of higher education for medicine, with almost 1.7 million students, and had more than 400,000 new graduates in 2008 [3]. Mostly because of its history and culture, medical education in China faces different problems than those in developed countries. Chinese teaching usually relies on rote learning and instruction instead of proactive investigation by students and promotion of creativity and imagination [4]. Therefore, the present challenge for both the government and medical schools is the improvement of the quality of medical education and training of qualified people who can both adapt to a rapidly changing world and simultaneously meet the needs of the Chinese people. Attempts have been made in some medical schools to modify teaching practices by including approaches based on clinical problems. Problem-based learning (PBL), with the aim of cultivating students’ creativity and practical abilities, has been adopted in China since the mid-1980s. While some recent studies have shown that skills of analysis and problem-solving ability have been improved among PBL students, the teaching of PBL is still new to many Chinese medical schools, and some participating scholars and instructors still have doubts about its educational benefits [3-6]. Therefore, new teaching methodology needs to be attempted and developed in China to meet the requirements of the current educational system for Chinese medical students.

The POPS system in immunology, pioneered by Parker A. Small, Jr., and Susan M. Johnson in the early 1970s at the University of Florida, Gainesville, permits students to work in small groups and solve clinical problems encountered in the field of immunology. The general purposes of POPS activities include helping students to learn how to apply basic science knowledge to the solution of clinical problems, facilitating students’ learning of how to better use sources (e.g., electronic databases, textbooks and peers) that will be available throughout their careers and encouraging students to work cooperatively [7]. This system is used extensively by medical, pharmacy, and other health profession schools throughout the United States and also other western countries, and many thousands of exercise booklets have been distributed free of charge by the Upjohn Company [8]. As compared to PBL, POPS requires the engagement of fewer teachers and can be more helpful for training clinical reasoning and clinical cooperation. Therefore, POPS could be more suitable for medical students than PBL in countries with limited teaching resources. In China and other developing countries, while PBL was introduced decades ago, the application of PBL is limited due to reasons like faculty shortage and lack of resources. Furthermore, a recent study among Indians pointed out that POPS could be a helpful alternative to PBL in developing countries [9]. Among Asians, many studies have been conducted on the application of POPS in various subjects in various forms [9-11].

While POPS could be a better option over the didactic lecture to teach undergraduate students, the application of POPS in China has never been reported, either in Chinese or in English, by the time we wrote this article. We report here the first trial of POPS in teaching immunology in China, and students’ and teachers’ perception of POPS was assessed as well.

## Methods

### Participants sampling

This study was conducted at the Institute of Immunology, Third Military Medical University of PLA, Chongqing, China. Recruits for this investigation were 321 second-year medical students and 16 teachers with good experience in teaching immunology.

### Design and instruments

**Figure 1:**
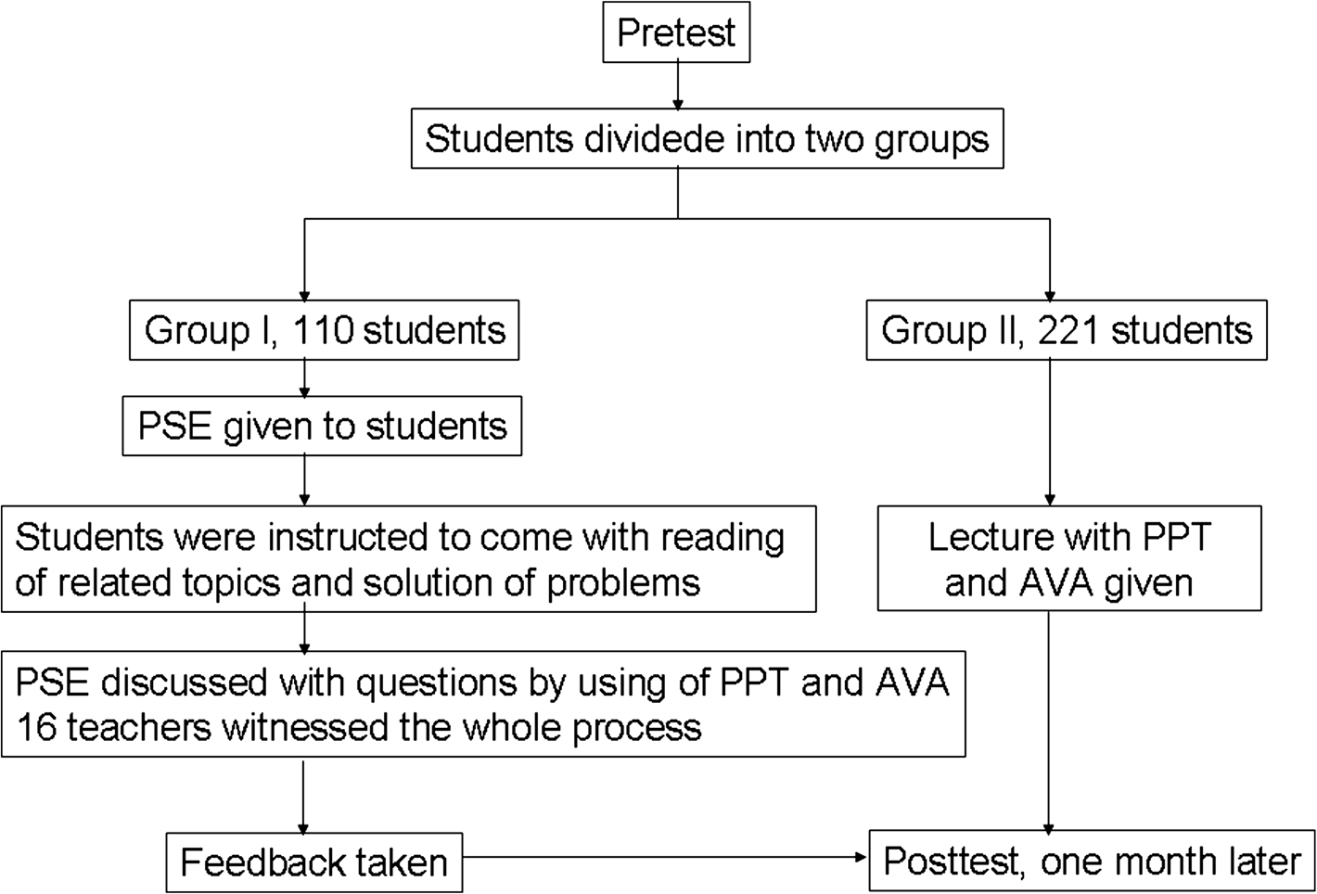
**Flow chart of POPS method.** Abbreviations: PSE, problem-solving exercise; PPT, PowerPoint presentation; AVA, audiovisual aids.

A total of 321 second-year students who are familiar with didactic lectures were divided into two groups: I and II. Group I, comprising 110 students, was taught by POPS, and Group II, including the remaining 211 students, was taught through traditional lectures. A POPS immunology teaching package to correlate basic and clinical sciences information about Immediate Hypersensitivity, which was originally developed by Parker A. Small, Jr., and Eric Brestel and was revised by Maru T. Fox, Wayne T. McCormack, Steven Specter and Gabriel Virella, was used in our POPS teaching (http://www.micro.musc.edu/pops/cases/HypersensitivityPOPS.pdf). The POPS exercise was performed strictly following the guide of the teaching package. An example of the POPS exercise is given in Appendix A. Sixteen teachers who had been lecturing to second-year medical students during the semester from February 11 to July 25, 2012, from the same institute or other departments were invited to read the teaching package and witnessed the teaching process. After teaching, a post-test with 10 different questions was administered to both groups (Figure 1).

### Measurements and data collection

A pre-test with 10 questions on the chapter of “immediate hypersensitivity” was performed for all students before class in order to evaluate their basic knowledge on immunology, while a post-test with 10 different questions on the same chapter was administered to both groups after class for the purpose of checking teaching effects. All questions were issued in the forms of single-choice and multiple-choice tests, and scoring was automatically done using a reading machine. These measures effectively minimize arbitrary judgments.

Later on, two self-structured questionnaire papers, which were adapted from Singh’s study, composed of 11 and 10 questions, respectively, were distributed to Group I students who were taught by POPS and all teachers who have audited the teaching process. The feedback forms were strictly anonymous [7]. All participants, 110 students and 16 teachers, completed the questionnaires and were included in this study. One week after the POPS trial, a discussion meeting was held among all involved teachers to summarize the merits, shortcomings and feasibility of the POPS teaching method in the university and anywhere else in China.

### Ethical review

The local Institutional Review Board at the Third Military Medical University waived ethics approval (application number: 20111028), as the study protocol was not deemed to represent bio-medical or epidemiological research, and no personal data were used. Procedures complied with data protection rules, and all data were anonymised prior to analysis.

### Statistical analysis

The differences between the pre- and post-test results of those in Groups I and II were compared by the *z* test using the software SPSS 17.0 for Windows (SPSS Inc., Chicago, USA) to the end of determining the statistical significance of the differences between Groups I and II.

## Results

### Demographic characteristics of participants

**Figure 2:**
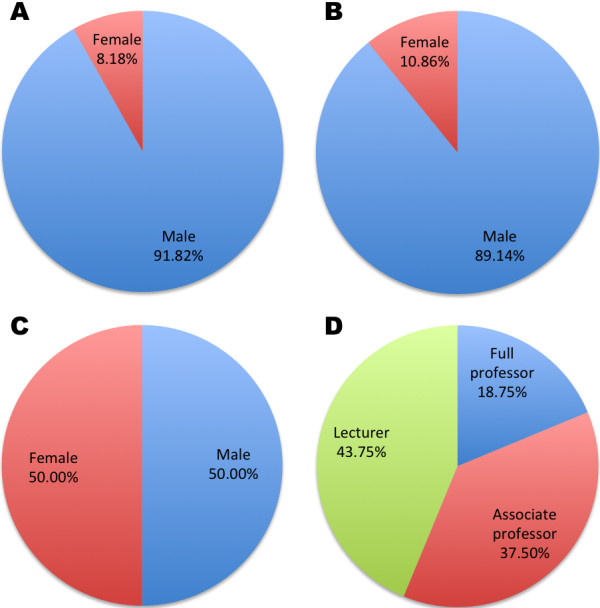
**Demographic characteristics of participants.** Gender percentage of 110 students in Group I (**A**), 221 students in Group II (**B**) and 16 teachers (**C**) as well as faculty position percentage of the teachers involved in this project (**D**).

Out of the 110 participating students in Group I, 101 were male and 9 were female, with a mean age of 20.5, while for the 221 students in Group II, 197 were male and 24 were female, with a mean age of 20.3 ± 0.42. Of the 16 involved teachers, 8 were male and 8 were female, and their mean age was 37.0 ± 6.34. According to their faculty positions, 3 were professors, 6 were associated professors and 7 were lecturers, and the mean teaching experience among them was 12 ± 6.34 years (Figure 2).

### Group differences in pre- and post-test scores

The mean scores and standard deviation (SD) of the pre-test for Groups I and II were 2.78 ± 0.67 and 2.35 ± 0.54, respectively, while the post-test results were 9.05 ± 1.25 and 5.53 ± 1.85 for the two groups, respectively. The intragroup differences in post- and pre-test in Groups I and II were 6.27 and 3.18, respectively. There was no statistically significant difference on the pre-test scores between both groups (*t* = 1.73, *P* > 0.05), but by contrast, considerable differences arose on the post-test scores between them (*t* = 4.85, *P* < 0.001) (Table 1). Notably, significant improvement in the mean difference in pre- and post-test scores of both groups was evaluated by *z* test (*z* = 15.74, *P* < 0.001), demonstrating the effectiveness of POPS teaching (*P* < 0.001) (Table 2).

**Table 1:** Pre- and post-test comparison between group I and II

	**Nubmer**	**Min**	**Max**	**Mean**	**SD**
Pre-test group I	110	0	6	2.78	0.67
Pre-test group II	221	0	6	2.35	0.54
				*t* =1.73	*P* > 0.05
Post-test group I	110	7	10	9.05	1.25
Post-test group II	221	4	8	5.53	1.85
				*t* =4.85	*P* < 0.001

**Abbreviations:***Min*, minimum; *Max*, maximum; *SD*, standard deviation; *t*, *t*-test.

**Table 2:** Paired difference correlations

	**Nubmer**	**Mean**	**SD**
Pre-test group I - Post-test group I	110	6.27	2.223
Pre-test group II - Post-test group II	221	3.18	1.864

**Notes:***z* = 15.74, *P* < 0.001 significant.

**Abbreviations:***SD*, standard deviation; *z*, *z*-test.

### Students’ perception of the POPS teaching

After the trial, the 110 students in Group I were asked to fill out a self-structured questionnaire anonymously. The statistic feedbacks are given in Table 3. Of the 11 questions answered by students, 85% of participants strongly agreed with 6 of them, and the remaining 5 had 50-85% of repliers who moderately agreed with them. No item generated a “disagree” response by more than 50% of students on the questionnaire. The student majority favored POPS-facilitated self-learning, reporting that it raised their interest in topic discussion, made them more absorbed in the class, strengthened their motivation to study and helped in keeping long-lasting memories and making diagnoses in real clinical practices. All of these results indicated that most students were in favor of the new teaching method and strongly preferred it to didactic lecturing. Notably, however, 25 (22.73%) students disagreed that POPS should be used by more teachers. In their opinion, not all teachers were suitable for POPS teaching.

**Table 3:** The POPS questionnaire answered by students

	**Questions**	**Highly agree**	**Moderate agree**	**Disagree**
1	Do you agree that POPS facilitates self-learning?	**45**	**59**	**6**
		40.91%	53.64%	5.45%
2	Do you agree that POPS should be used by more teachers?	**36**	**49**	**25**
		32.73%	44.55%	22.73%
3	Do you agree that POPS helps you to make diagnoses in real clinical practice?	**55**	**47**	**8**
		50.00%	42.73%	7.27%
4	Do you agree that self-reading ahead of the class benefits material understanding?	**57**	**49**	**4**
		51.82%	44.55%	3.64%
5	Do you agree that POPS raises your interest in topic discussion?	**41**	**57**	**12**
		37.27%	51.82%	10.91%
6	Do you agree that POPS make you more absorbed during the class?	**47**	**45**	**18**
		42.73%	40.91%	16.36%
7	Do you agree that POPS is a more scientific way for medical teaching?	**41**	**50**	**19**
		37.27%	45.45%	17.27%
8	Do you agree that POPS strengthens your intrinsic motivation for studying?	**39**	**52**	**19**
		35.45%	47.27%	17.27%
9	Do you agree that POPS develops your self-directed learning skills?	**48**	**53**	**9**
		43.64%	48.18%	8.18%
10	Do you agree that POPS provides systemic approaches in applying findings of cognitive psychology to the educational process?	**43**	**53**	**14**
		39.09%	48.18%	12.73%
11	Do you agree that POPS provides benefits in terms of long-lasting memory?	**45**	**56**	**9**
		40.91%	50.91%	8.18%
	**Average (%)**	**45.18**	**51.82**	**13**
		41.07%	47.11%	11.82%

### Teachers’ perception of the POPS teaching

Sixteen teachers who had rich experience in teaching immunology were invited to audit the POPS teaching process and were subsequently asked to anonymously complete a similarly structured questionnaire. Compared with the questionnaire answered by students, two questions concerning students’ personal awareness were deleted while one question requesting the teachers’ opinion of applying POPS in medical education was added in the one designed for teachers. The statistic results were given in Table 4. Similarly, out of the 10 questions answered by teachers, 7 generated agreement by over 85% of responders, and 2 questions demonstrated moderate agreement by 75% of responders; however, the last question had striking disagreement by 75% of the teachers. The positive responses suggested that most teachers had similar opinions on the effects of POPS, including raising students’ interest, retaining their attention, enhancing their intrinsic motivation and giving systemic approaches in applying findings of cognitive psychology to the educational process. As expected, all teachers thought that self-reading ahead of the class did help in teacher-student interaction, since POPS is a clinical problem-driven teaching method in itself and hence requires pre-reading for better understanding of the topic in question.

**Table 4:** The POPS questionnaire answered by teachers

	**Questions**	**Highly agree**	**Moderate agree**	**Disagree**
1	Do you agree that POPS facilitates students’ self-learning?	**5**	**11**	**0**
		31.25%	68.75%	0.0%
2	Do you agree that POPS helps medical students to make diagnoses in real clinical practice?	**2**	**10**	**4**
		12.5%	62.5%	25%
3	Do you agree that self-reading ahead of class helps in teacher-student interaction?	**9**	**7**	**0**
		56.25%	43.75%	0.0%
4	Do you agree that POPS raises students’ interest in teaching topics?	**5**	**10**	**1**
		31.25%	62.5%	6.25%
5	Do you agree that POPS helps keep students’ attention during the class?	**7**	**7**	**2**
		43.75%	43.75%	12.5%
6	Do you agree that POPS is a more scientific way of teaching than lecturing?	**1**	**11**	**4**
		6.25%	68.75%	25%
7	Do you agree that POPS strengthens students’ intrinsic motivation?	**3**	**12**	**1**
		18.75%	75%	6.25%
8	Do you agree that POPS develops self-directed learning skills?	**3**	**12**	**1**
		18.75%	75%	6.25%
9	Do you agree that POPS gives systemic approaches in applying findings of cognitive psychology to educational process?	**1**	**13**	**2**
		6.25%	81.25%	12.5%
10	Do you agree that POPS is a suitable method for current medical education in China?	**1**	**3**	**12**
		6.25%	18.75%	75%
11	**Average (%)**	**3.70**	**9.60**	**2.70**
		23.12%	60%	16.88%

Still, 25% of the teachers doubted whether POPS was a more scientific way of teaching than didactic lecturing, and more negatively, 75% of them disagreed that POPS is a suitable teaching method for the current medical education system in China. With regard to this question, all teachers were asked to give reasons for their choices in the following discussion meeting (see below).

### Teachers’ opinions on the feasibility of POPS in China

A discussion meeting was held shortly after the POPS teaching trial was held, and all teachers who had audited the process were invited to comment on this trial. In contrast to the satisfactory response from the students, most teachers stated their concerns from more practical perspectives. Their comments are summarized in the following aspects:

1. Limited teaching resources (e.g. inadequate space, insufficiently available references and shortage of teachers with enough experience of POPS or PBL) hinder wide application of POPS in many Chinese universities;

2. Preparation of POPS teaching is too time consuming for teachers in medical universities who have to spend a great deal of time on research work;

3. The pre-reading request in POPS greatly increases the burden of those students whose curricula are often much heavier in Asian countries than of those in other regions worldwide.

## Discussion

The traditional teaching method in China is lecture-based learning, which requires teachers to give didactic lectures strictly following the rationales on textbooks. As a consequence of this fossilized teaching mode, the extent of students’ curiosity and motivation mostly depends on the quality of teacher-centered presentations [12-14]. In contrast to lecture-based learning, PBL is an innovative technique introduced in medical education since 1969 [15], and has been widely used in the curriculum in worldwide medical schools, especially in the western countries [16,17]. The PBL approach was first introduced in China in 1986, and since then it has been increasingly applied in many Chinese medical universities [18]. In PBL teaching trials, the students learned to use various sources of information effectively and are trained in the rapid retrieval of relevant knowledge. These skills are important for medical professionals who would deal with clinical problems in reality. As an alternative of PBL, POPS has never been attempted in medical education in China, and therefore our trial reported here was the first case.

In this study, POPS teaching was used in medical immunology, which is an important discipline in modern medical education. The effect of POPS teaching was compared with traditional lecturing. Although there was no significant difference on the pre-test between the two student cohorts, the difference on the post-test was statistically great (Table 1). The more pronounced difference between the pre-test and post-test performance in the POPS group than that of the non-POPS group formed sharp contrast (Table 2), which indicated that the students taught by POPS had made greater progress in their knowledge structure after learning. These results clearly showed that the students taught by POPS had acquired better skills in gathering information, linking new information with existing data and expressing thoughts and ideas, which was also exhibited in the students’ perception (Table 3). More importantly, the majority of students confessed that POPS raised their interest in topic discussion, and they thus preferred POPS to didactic lectures.

Despite the striking advantages in PBL or POPS over traditional lecturing, there have been many doubts regarding whether these student-centered teaching methods are feasible in China or other Asian countries, since oriental philosophy and culture are remarkably different from those in western countries. As a matter of fact, some Chinese educationists have studied this question and concluded that appropriate modifications on PBL or POPS techniques were required in order to fit for the specific educational environment in China [14,18,19].

According to their study, the biggest hindrance for widespread application of PBL is the shortage of medical teachers. This problem has become more intense since 1999, when a new governmental policy encouraging universities to enroll more students took effect. As a consequence of this policy, the number of enrolled students in all of China’s universities rapidly increased from 4.09 million in 1999 to 20.2 million in 2008, but the teacher-student ratio decreased from 1:14 to 1:17 over the past decade as the staff increase failed to keep pace with the student increase [20]. The ratio for undergraduate education in medical universities may be even larger (i.e. approaching 20 in the third military medical university, which is 4–5 times higher than the figure in USA [20]). Since both PBL or POPS teaching involve teaching students in small groups under the supervision of tutors, a sufficient number of qualified teachers is the prerequisite of using these techniques. Therefore, modifications that require fewer teachers need to be introduced to medical universities in populous countries; e.g. China and India.

Another notable adverse fact in popularizing PBL or POPS lies in the textbooks used for undergraduate students in China [14,19]. Owing to the long-term application of didactic lectures and the distinctive linguistic environment, almost all textbooks were prepared for LBL and written in Chinese. No textbooks specifically prepared for PBL or POPS is available in Chinese. As pre-reading is crucial for POPS teaching, several references written in English may need to be translated into the native language of medical students in China; otherwise, they have to spend much more time on pre-reading than those students who are native speakers of English. Still, this situation has been gradually alleviated because of the continuously improved English-speaking skills of Chinese students and extensive application of online and other various resources in medical teaching.

### Limitations

We believe that our study has the following limitations:

1. The conclusions drawn from a single trial may be enhanced by more trials and a longer time study.

2. The study was limited to one system of teaching in immunology.

3. The POPS teaching package used in this trial was developed by American educationists, which might not be appropriate for medical students in China or other Asian countries.

4. Lecture quality affected by personal styles may not be eliminated, since the two student cohorts were taught by different teachers.

## Conclusions

The results of our trial clearly showed that POPS was an efficient teaching method for immunology education and was preferred by students to didactic lectures. Teachers who witnessed the teaching process also demonstrated positive responses to POPS. However, concerns about the feasibility of POPS in China arose among teachers, in consistence with published studies. Some modifications on POPS may be needed to fit the educational environment in China.

## Appendix A

### An example of POPS and related questions

Harry Hoofit, an outdoorsman whose main hobby is hiking, has just come to your office. This morning, while alone on a nearby trail, he was stung on the left forearm by an unknown species of insect. He felt immediate pain and his arm began to swell, but he had been bitten many times before and therefore paid no attention to the sting. Within a few minutes, however, he became very apprehensive, became short of breath, and experienced increasing difficulty in breathing until he passed out. When he awoke, his entire arm was markedly swollen and difficult to move because of the swelling. He had hives (raised, white and itchy blotches) all over his body. He is not sure how long he was unconscious but believes it was at least ten minutes but not more than one hour. He also discovered that his underwear and pants were wet; he thinks he urinated while unconscious. He is naturally concerned and wants to know three things:

1) What caused this frightening event?

2) Might it happen again?

3) If so, can you help him?

Q1. The wheal of a wheal-and-flare reaction

a. occurs 24–72 hours post-injection.

b. is caused by edema resulting from the histamine-induced capillary permeability.

c. is caused by vasodilatation and increased blood flow.

d. can be induced by haptens.

e. None of the above.

Q2. A "RAST" assay

a. is usually used to determine the amount of blocking antibody in a patient's serum.

b. proves what allergen is causing a patient's allergy.

c. requires an insolubilized allergen and radiolabeled anti-IgE.

d. is the "ragweed allergy standard test."

e. gives the same information as a skin test.

Q3. Which of the following substances stabilizes mast cell membranes and thereby reduces release of histamine from mast cells?

a. Cromolyn sodium

b. Antihistamines

c. Epinephrine

d. Blocking antibody

e. None of the above

Q4. The principal difference between asthma and allergic rhinitis is that

a. asthma occurs year round, and allergic rhinitis occurs only in late summer.

b. asthma is an allergy, and allergic rhinitis is psychogenic.

c. asthma affects mostly females, and allergic rhinitis affects mostly males.

d. asthma affects the lower respiratory tract, whereas allergic rhinitis affects the upper respiratory tract.

e. asthma can be diagnosed with skin tests, but allergic rhinitis must be diagnosed with the RAST assay.

Q5. Which of the following substances inhibits allergic disease by preventing the antigen from reaching the reagenic antibody fixed to the mast cell?

a. Cromolyn sodium

b. Antihistamines

c. Epinephrine

d. Blocking antibody

e. None of the above

Q6. Which of the following cytokines is believed to be the primary determinant of a vigorous IgE response to an allergen?

a. IL-2

b. IL-4

c. IL-5

d. IL-12

e. IL-13

Q7. Mast cells and basophils are very similar in that they both

a. have receptors on their surfaces that bind the Fc region of IgM.

b. are found in the blood.

c. have granules that contain histamine.

d. stain with acidophilic dyes.

e. synthesize antibodies.

Q8. A patient who is allergic to ragweed developed IgE myeloma. The myeloma IgE does not react with the ragweed pollen. What would be the effect of his myeloma on the severity of his allergic symptoms during hay fever season?

a. No change.

b. It would increase due to his having more circulating IgE.

c. It would increase due to the blocking effect of the myeloma.

d. It would decrease due to competitive inhibition of IgE anti-ragweed binding to mast cell receptor sites by myeloma IgE.

e. It would decrease due to competitive inhibition of IgE anti-ragweed binding to ragweed allergen by myeloma IgE.

Q9. The usual sequence of events in an allergic reaction is as follows:

a. the allergen combines with circulating IgE and then the IgE:allergen complex binds to mast cells.

b. the allergen binds to the IgE already fixed to mast cells.

c. the IgE binds allergen in the blood and then binds to histamine receptors.

d. the allergen is processed by macrophage and then binds to mast cells.

e. the allergen combines with IgG.

Q10. Complement

a. is never involved in allergic reactions.

b. can be fixed by IgE antibody-antigen complexes.

c. can produce anaphylaxis by release of anaphylotoxin (C3a and C5a) when complement is fixed.

d. is involved in allergic rhinitis.

e. can lyse mast cells releasing IgE.

## Abbreviations

PBL: Problem-based learning; POPS: Patient-oriented problem-solving system; SD: Standard deviation.

## Competing interest

The authors declare no competing interests.

## Authors’ contributions

ZZ, WL and YW were responsible for the study concept and design. SG and JH contributed to teaching, data collection and data analysis. ZZ and WL prepared the manuscript. YW supervised the whole investigation process. All authors read and approved the final manuscript.

## Pre-publication history

The pre-publication history for this paper can be accessed here:

http://www.biomedcentral.com/1472-6920/13/11/prepub
